# Feline coronavirus replication is affected by both cyclophilin A and cyclophilin B

**DOI:** 10.1099/jgv.0.000663

**Published:** 2017-03-13

**Authors:** Yoshikazu Tanaka, Yuka Sato, Takashi Sasaki

**Affiliations:** ^1^​ Department of Veterinary Hygiene, Veterinary School, Nippon Veterinary and Life Science University, 1-7-1 Kyounan, Musashino, Tokyo 180-8602, Japan; ^2^​ Division of Molecular Virology, Department of Microbiology and Immunology, The Institute of Medical Science, The University of Tokyo, 4-6-1, Shirokanedai, Minato-ku, Tokyo 108-8639, Japan; ^3^​ Department of Microbiology, Faculty of Medicine, Juntendo University, Tokyo 113-8421, Japan

**Keywords:** feline coronavirus, peptidyl-prolyl *cis-trans* isomerase, cyclophilin, feline infectious peritonitis

## Abstract

Feline coronavirus (FCoV) causes the fatal disease feline infectious peritonitis, which is currently incurable by drug treatment, and no effective vaccines are available. Cyclosporin A (CsA), a cyclophilin (Cyp) inhibitor, inhibits the replication of FCoV *in vitro* and *in vivo* as well as the replication of human and animal coronaviruses. However, the mechanism underlying the regulation of coronavirus replication by CsA is unknown. In this study, we analysed the role of Cyps in FCoV replication using knockdown and knockout cells specific to Cyps. Inhibition of CypA and CypB reduced FCoV replication, with replication in knockout cells being much less than that in knockdown cells. Furthermore, the proteins expressed by CypA and CypB harbouring mutations in their respective predicted peptidyl-prolyl *cis–trans*isomerase active sites, which also alter the affinities between Cyps and CsA, inhibited FCoV replication. These findings indicate that the peptidyl-prolyl *cis–trans*isomerase active sites of Cyps might be required for FCoV replication.

## Introduction

*Feline coronavirus* (FCoV) is an enveloped, positive-stranded RNA virus of the *Coronaviridae* family [1]. Coronaviruses (CoVs) cause severe diseases of the respiratory system, gastrointestinal tract and central nervous system [2]. FCoV causes feline infectious peritonitis (FIP), which is a transmissible inflammation of the visceral serosa and omentum with exudation into the abdomen [3]. Another form of the disease is characterized by granulomatous involvement of parenchymatous organs such as the kidneys, mesenteric lymph nodes, bowel wall, liver, central nervous system and eyes [4]. Mortality is extremely high once clinical signs appear, and no effective drugs for treating FIP are available.

Viral replication requires numerous host factors [5, 6] that represent potential targets of antiviral therapy. RNA viral genomes typically replicate with low fidelity and undergo rapid evolutionary changes. Therefore, inhibitors targeting host factors may be preferred as the development of resistance is less likely. Cyclophilins (Cyps) and FK506-binding proteins belong to peptidyl-prolyl *cis–trans* isomerase (PPIase) families that catalyse *cis–trans* isomerization of the imidic prolyl peptide bond and are pharmacological targets of cyclosporin A (CsA) and FK506 [7, 8]. CsA is a well-known immunosuppressive drug that binds to cellular Cyps to inhibit calcineurin, a calcium–calmodulin-activated serine/threonine-specific phosphatase. The inhibition of calcineurin blocks the translocation of the nuclear factor of activated T cells from the cytosol into the nucleus, thus preventing the transcription of genes encoding cytokines, such as interleukin-2. CsA is a potent inhibitor of certain human and animal CoVs [9, 10]. However, FK506 also suppresses calcineurin activity and signalling through the nuclear factor of activated T cells at the same stage as CsA but without affecting FCoV replication [11]. Moreover, the replication of animal CoVs such as infectious bronchitis virus and transmissible gastroenteritis virus is not inhibited by FK506 [12]. However, this drug effectively inhibits the replication of human CoVs, severe acute respiratory syndrome CoV (SARS-CoV), HCoV-NL63 and HCoV-229E [13]. This indicates that the mechanism underlying CoV replication between human and animals requires different cellular immunophilins such as Cyps and FK506-binding proteins. The mechanism underlying the inhibitory effects of CsA on CoV replication is poorly understood, although the fact that CypA and CypB do not affect SARS-CoV replication [9] is important. Furthermore, HCoV-NL63 replication depends on CypA but not CypB [14]. In addition, single-nucleotide polymorphism variants near the human CypA active site are required for HCoV-229E replication [12].

To investigate the roles of CypA and CypB in CoV replication, we inhibited their expression using RNA-silencing techniques, CRISPR/Cas9 knockout (KO) and host cells transfected with PPIase active-site mutants of CypA and CypB. Our study indicates that the PPIase active sites of CypA and CypB, which alter the affinities between Cyps and CsA, might be required for CoV replication.

## Results

### Cyps promote viral replication

**Fig. 1.**
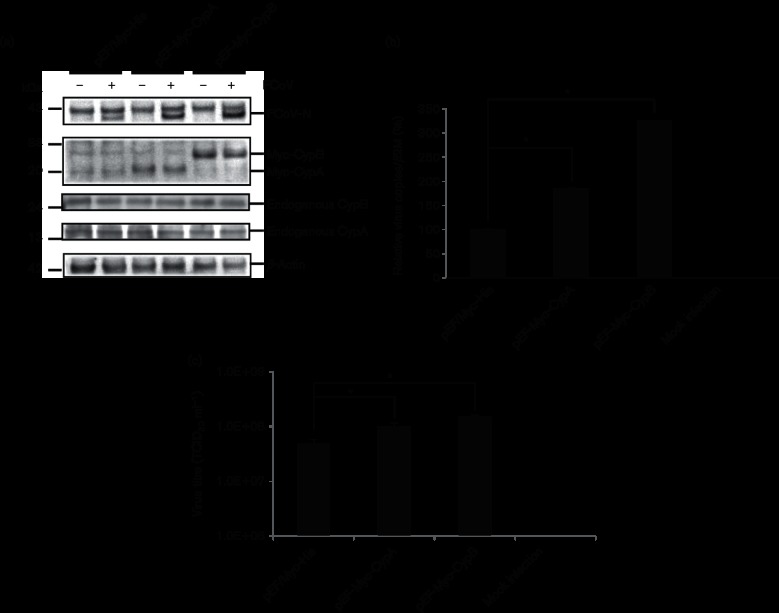
Stable expression of CypA and CypB enhances FCoV replication. (a) The *Felis catus* whole fetus-4 cell line was transfected with pEF-Myc-CypA, pEF-Myc-CypB or the pEF/Myc-His vector (empty vector), and the transfected cells were selected with blasticidin at 24 h after transfection. The individual cell colonies selected with blasticidin were cloned. The cells were infected with FCoV, and lysates were subjected to Western blot with anti-Myc, anti-FCoV-N, anti-CypA, anti-CypB and anti-β-actin antibodies, respectively. FCoV-N protein expression was normalized to that of endogenous β-actin. All strips were excised from the same blot. (b) RT-qPCR analysis of FCoV-infected cells stably expressing Myc-CypA and Myc-CypB was performed to determine viral copies. Total RNAs were extracted and analysed by RT-qPCR. Virus copies were normalized with β2-microglobulin (*β2M*) gene expression. Experiments were performed in triplicate. (c) Cells stably expressing Myc-CypA or Myc-CypB were infected with FCoV. The viral titration with the supernatants of the infected or mock-infected cells was determined by TCID_50_ assay. Error bars indicate the sd from the mean in (b) and (c). Asterisks indicate significant differences (*P<0.05) in (b) and (c).

Western blot analysis revealed that, compared with controls, cells transfected with Myc-tagged *CypA* or *CypB* showed increased viral replication (Fig. 1a). This was consistent with the results of reverse transcriptase-quantitative PCR (RT-qPCR) assays that showed 1.8- and 3.2-fold increases of FCoV-N gene in the cells stably expressed with *CypA* and *CypB*, respectively, compared with controls (Fig. 1b). Numbers of viral progeny in cells stably expressed with *CypA* and *CypB* were 2.0- and 3.0-fold higher than those in controls, respectively (Fig. 1c). Both transfected *Cyp* genes included cMyc tag and 6× histidine tag at the carboxyl-terminal sites. Therefore, the present molecular mass of both Cyps was approximately 5000 Da more than that the original Cyp proteins.

### Knockdown of CypA or CypB expression inhibits FCoV replication

**Fig. 2.**
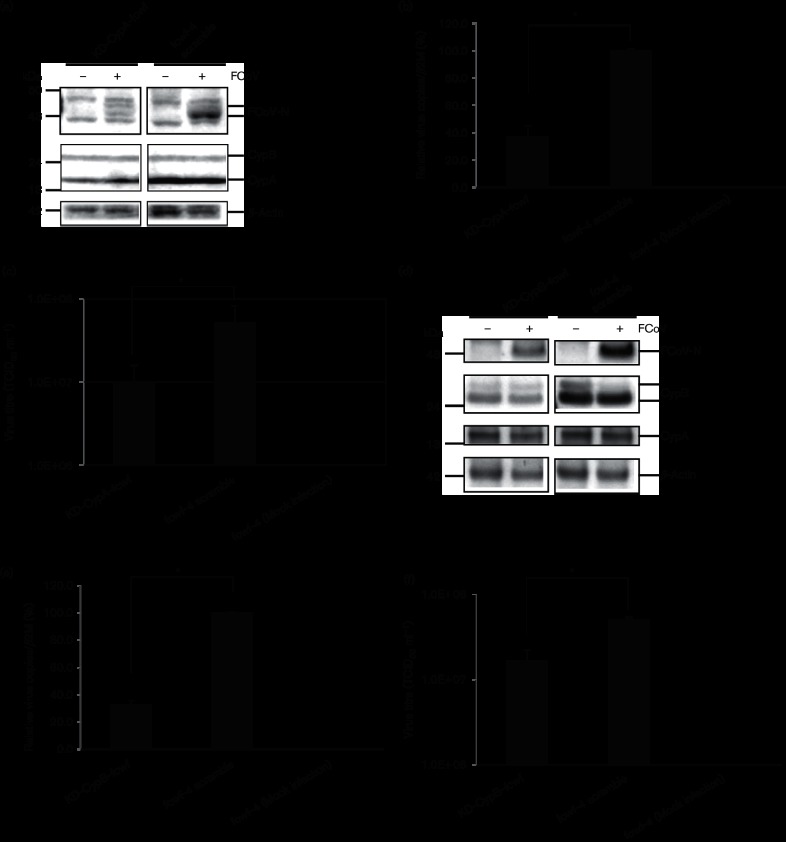
CypA and CypB play important roles in the replication of FCoV. (a) KD-CypA-fcwf and fcwf-4 cell lines transfected with a non-targeting (scramble) shRNA vector (as a control) were infected with FCoV. After 20 h infection, the cells were disrupted with lysis buffer and used for Western blot analysis with anti-CypA, anti-CypB, anti-FCoV-N and anti-β-actin antibodies. FCoV-N protein was normalized with endogenous β-actin protein. The three strips were excised from the same blot. (b) Total RNAs from infected or mock-infected cells were extracted and analysed by RT-qPCR. RT-qPCR was performed to determine viral copies. The fcwf-4 cell line was transfected with a non-targeting (scramble) shRNA vector as a control. Experiments were performed in triplicate. Viral copies were normalized to those of feline *β2M*. (c) FCoV replication in KD-CypA-fcwf cells was analysed by determining TCID_50_ using supernatants of the infected cells. The fcwf-4 cell line transfected with a non-targeting shRNA vector was used as a control. (d) Western blot analysis of the KD of endogenous CypB expression using shRNA and the expression of FCoV-N in FCoV-infected cells. Western blot with anti-CypA, anti-CypB, anti-FCoV-N and anti-β-actin antibodies was performed. Expression levels were normalized to that of β-actin. All strips were excised from the same blot. (e) Total RNAs from infected or mock-infected cells were extracted and analysed using RT-qPCR. RT-qPCR was performed to determine viral copies. The fcwf-4 cell line was transfected with a non-targeting (scramble) shRNA vector as a control. Experiments were performed in triplicate. Viral copies were normalized to those of feline *β2M*. (f) FCoV replication in KD-CypB-fcwf cells was analysed by determining TCID_50_. The fcwf-4 cell line transfected with a non-targeting shRNA vector was used as a control. Viral titration with the supernatants of the infected cells was determined by TCID_50_ assay. Experiments were performed in triplicate. The error bars in (b), (c), (e) and (f) indicate the sd from the mean. Asterisks indicate significant differences (*P<0.05) in (b), (c), (e) and (f).

To determine how CypA and CypB contribute to FCoV replication, we established cells that expressed short hairpin RNAs (shRNAs) specific to *CypA* and *CypB*. Decreases in the levels of expression of CypA or CypB had a slight effect on cell growth but not on cell viability (data not shown). Two bands of FCoV-N revealed in Western blot analysis were indicative of phosphorylated and unphosphorylated forms. CypA expression decreased by approximately 80 % in cells expressing *CypA* shRNA compared with control cells [*Felis catus* whole fetus-4 (fcwf-4) cell line scramble] transfected with non-targeting shRNA (Fig. 2a). Furthermore, FCoV-N expression in FCoV-infected knockdown (KD)-CypA-fcwf cells was decreased (Fig. 2a), and the numbers of viral copies and progeny produced by the KD-CypA-fcwf cells were reduced by approximately 65 and 80 %, respectively (Fig. 2b, c).

KD of *CypB* reduced viral protein expression and the number of viral copies by approximately 70 % compared with controls (Fig. 2d, e). The number of viral progeny in the KD-CypB-fcwf cells was reduced by approximately 70 % (Fig. 2f). The expression of a larger isoform of CypB in Fig. 2(d) may be the uncleaved or glycosylated form of CypB as described by Spik *et al.* [15]. These data suggest that the replication of the FCoV and production of virus progeny requires host CypA and CypB.

### Enforced expression of CypA and CypB in KD cells restores viral replication

**Fig. 3.**
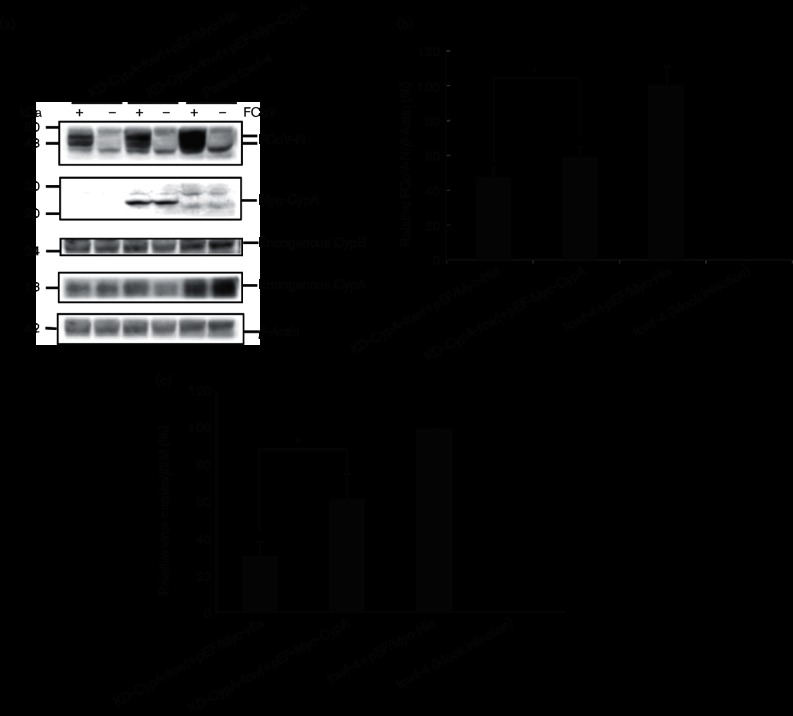
KD-CypA cells transfected with wild-type CypA restore viral replication. (a) KD-CypA-fcwf cells transfected with pEF-Myc-CypA were infected with FCoV, and expression of FCoV-N was analysed by Western blot analysis using anti-FCoV-N, anti-Myc, anti-CypA, anti-CypB and anti-β-actin antibodies. FCoV-N levels were normalized to that of endogenous β-actin. The five strips were excised from the same blot. (b) Western blot analysis of FCoV-N expression. KD-CypA-fcwf cells were transfected with pEF/Myc-His (empty vector) or pEF-Myc-CypA, and the parental fcwf-4 cells were transfected with the pEF/Myc-His (empty vector). Experiments were performed in triplicate, with error bars indicating the sd from the mean. (c) RT-qPCR was performed to determine viral copies of KD-CypA cells stably expressing Myc-CypA, infected with FCoV. Total RNAs from infected or mock-infected cells were extracted and analysed by RT-qPCR. Viral copies were normalized to those of the feline *β2M*. Experiments were conducted in triplicate, and error bars indicate the sd from the mean. Asterisks indicate significant differences (*P<0.05) in (b) and (c).

**Fig. 4.**
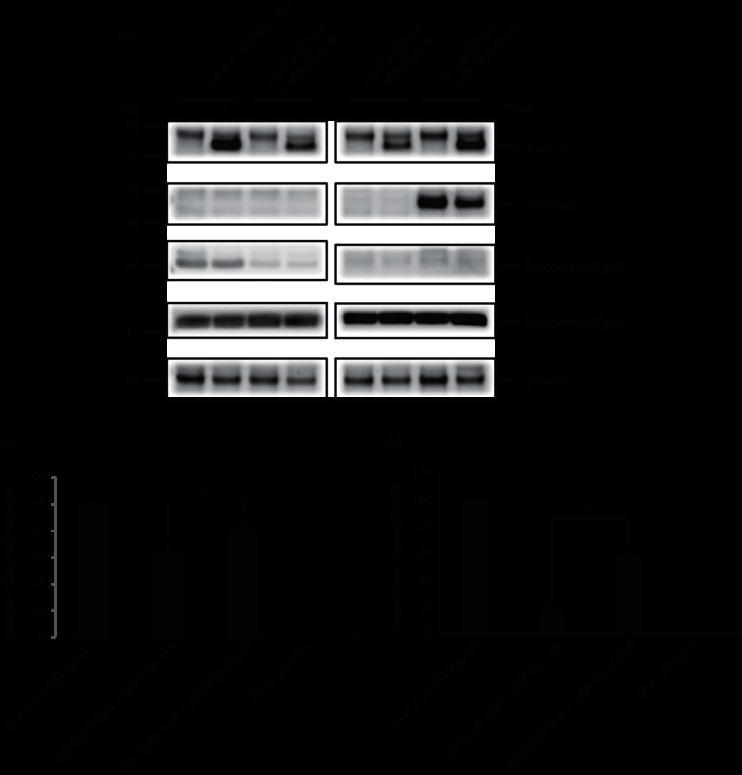
KD-CypB cells transfected with pEF-Myc-CypB support viral replication. (a) KD-CypB-fcwf cells transfected with pEF-Myc-CypB and the pEF/Myc-His (empty vector), respectively, were infected with FCoV. Parental fcwf-4 cells were transfected with pEF/Myc-His. Lysates were analysed by Western blot using anti-FCoV-N, anti-Myc, anti-CypB, anti-CypA and anti-β-actin antibodies, respectively. All strips were excised from the same blot, and protein expression levels were normalized to that of β-actin. (b) Western blot analysis of the effect of CypB restored with pEF-Myc-CypB on FCoV-N expression. FCoV-N expression was normalized to that of β-actin. (c) RT-qPCR was performed to determine viral copies of KD-CypB cells stably expressing Myc-CypB infected with FCoV. Total RNAs from infected or mock-infected cells were extracted and analysed using RT-qPCR. Viral copies were normalized to those of the feline *β2M*. Experiments were conducted in triplicate, and error bars indicate the sd from the mean. Asterisk indicates significant differences (*P<0.05) in (b) and (c).

To determine whether *CypA* and *CypB* shRNAs inhibited Cyp expression, Myc-tagged wild-type *CypA* and *CypB* were expressed in KD-CypA-fcwf and KD-CypB-fcwf cell lines, respectively. Cells transfected with pEF-Myc-CypA and pEF-Myc-CypB were infected with FCoV. Compared with KD-CypA-fcwf cells, viral protein expression and genome copy number were restored in cells transfected with *CypA* (Fig. 3a–c). Expression of FCoV-N in KD-CypB-fcwf cells transfected with *CypB* was approximately 15 % higher than that in KD-CypB-fcwf cells (Fig. 4a, b). Moreover, the number of viral copies in the KD-CypB-fcwf cells transfected with *CypB* was restored by approximately 30 % compared with that in the KD-CypB-fcwf cells (Fig. 4c). This demonstrates the specificities of KD-CypA and KD-CypB and that viral replication requires CypA and CypB.

### FCoV replication is inhibited in CypA- or CypB-KO cell lines

**Fig. 5.**
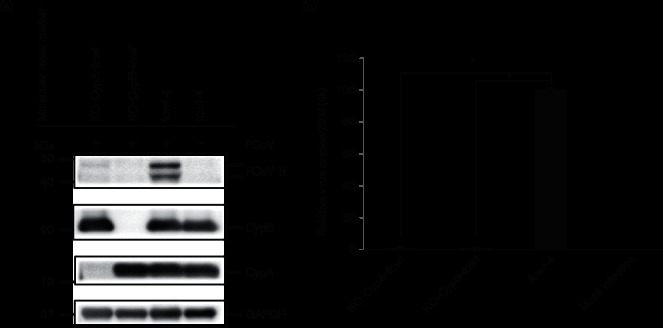
FCoV replication is inhibited in KO-CypA-fcwf and KO-CypB-fcwf cells. (a) Stable KO cells for *Cyp* genes, *CypA* and *CypB*, were infected with FCoV. At 20 h post-infection (p.i.), total cell lysates were analysed by Western blot using anti-FCoV-N, anti-CypA, anti-CypB and anti-glyceraldehyde 3-phosphate dehydrogenase (GAPDH) antibodies. All strips were excised from the same blot, and FCoV-N protein expression levels were normalized to those of GAPDH. (b) Total RNAs were extracted for RT-qPCR analysis at 20 h p.i. after each KO-Cyps cell line was infected with FCoV at a m.o.i. of 1. Viral copies were normalized to those of the feline *β2M*. Experiments were conducted in triplicate, and error bars indicate the sd from the mean. Asterisk indicates significant differences (*P<0.05).

When FCoV-infected fcwf-4 cells were transfected with shRNAs against *CypA* and *CypB*, the protein expression levels of FCoV-N were reduced but not completely inhibited in fewf-4 cells. Therefore, to further examine the role of *CypA* and *CypB*, we conducted genetic KO experiments in fcwf-4 cells using CRISPR/Cas9 systems. We confirmed protein expression levels of *CypA* and *CypB* by Western blot analysis, which showed that the protein expression of *CypA* and *CypB* was almost undetectable (Fig. 5a). Western blot and RT-qPCR analyses showed that viral replications in the CypA-KO and CypB-KO cells infected with FCoV were reduced by more than 95 % compared with those in the infected parent fcwf-4 cells (Fig. 5b), indicating that both CypA and CypB proteins significantly contribute to FCoV replication.

### The putative active sites of Cyp isomerases reduce FCoV replication

**Fig. 6.**
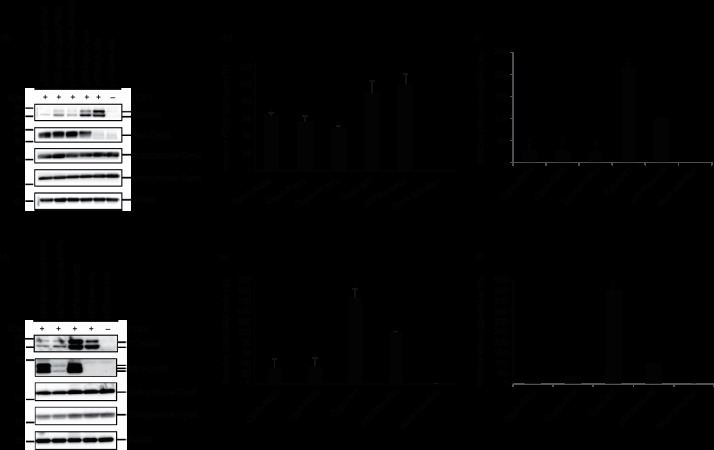
PPIase active sites of CypA and CypB are involved in FCoV replication. (a) fcwf-4 cells transfected with vectors, which express Myc-tagged CypA mutants (CypA-R55A, CypA-R60A and CypA- H126Q), were infected with FCoV. Cell lysates were prepared for Western blot analysis at 20 h p.i. The transferred membrane was incubated with anti-FCoV-N, anti-Myc, anti-CypA, anti-CypB and anti-β-actin antibodies. FCoV-N expression levels were normalized to that of β-actin, and all strips were excised from the same membrane. (b) Western blot analysis of the effect of CypA mutants on FCoV-N expression. FCoV-N protein expression levels were normalized to that of β-actin. (c) Total RNAs were extracted for RT-qPCR analysis at 20 h p.i. after the cells transfected with each vector encoding a dominant-negative gene were infected with FCoV at a m.o.i. of 1. Viral copies were normalized to those of the feline *β2M*. (d) fcwf-4 cells transfected with vectors, which express Myc-tagged CypB mutants (CypB-R62A and CypB-F67A), were infected with FCoV, and cell lysates were prepared for Western blot analysis at 20 h p.i. The transferred membrane was incubated with anti-FCoV-N, anti-Myc, anti-CypA, anti-CypB and anti-β-actin antibodies. FCoV-N expression levels were normalized to that of β-actin, and all strips were excised from the same membrane. (e) Western blot analysis of the effect of CypB mutants on FCoV-N expression. FCoV-N protein expression levels were normalized to that of β-actin. (f) Total RNAs were extracted for RT-qPCR analysis at 20 h p.i. after the cells transfected with each vector encoding the dominant-negative gene were infected with FCoV at a m.o.i. of 1. Viral copies were normalized to those of the feline *β2M*. Experiments were conducted in triplicate, and error bars indicate the sd from the mean. Asterisk indicates significant differences (*P<0.05) in (b), (c), (e) and (f).

We examined the effects of the proteins expressed by *CypA* harbouring mutations in their respective predicted PPIase active sites (Table S1, available in the online Supplementary Material). We replaced Arg-55, Phe-60 and His-126 in the PPIase catalytic domain of CypA with Ala (R55A-CypA and F60A-CypA mutants) and Gln (H126Q-CypA mutant) (Table S1). These three sites are strictly conserved in many species and might be dominant-negative inhibitors of isomerase activity [16]. Moreover, these sites drastically reduce the affinities between CypA and CsA as shown by moe (Molecular Operating Environment) software analysis (Fig. S1). fcwf-4 cells were transfected with CypA mutants (CypA-R55A, CypA-F60A and CypA-H126Q), and all mutant proteins were expressed (Fig. 6a) as predicted in Fig. S1. FCoV-N expression decreased in the mutant-expressed cells after infection with FCoV (Fig. 6b). Protein expression levels of endogenous *CypA* and *CypB* were not affected by the mutant expression (Fig. 6a). However, compared with empty-vector-transfected cells, wild-type CypA did not inhibit FCoV replication. Although the number of virus copies in the cells transfected with wild-type *CypA* was increased, those in all cells transfected with the mutant genes were reduced compared with control cells (Fig. 6c).

Next, we considered whether the respective predicted PPIase active sites of CypB promote FCoV replication. Therefore, we replaced Arg-62 and Phe-67 in the PPIase catalytic domain of CypB with Ala (R62A-CypB mutant) and Ala (F67A-CypB mutant) (Table S2). These sites are conserved in many species. Carpentier *et al*. [17] reported that these residues lead to a dramatic loss of PPIase activity, approximating 8–12 % of wild-type CypB isomerase activity. Also, these two sites drastically reduce the affinity between CypB and CsA as shown by moe software analysis (Fig. S2). Although the protein expression level of CypB-F67A mutant was less than that of the CypB-R62A mutant as predicted in Fig. S2, FCoV-N expression levels were reduced by approximately 70 % in the cells transfected with plasmids encoding each mutant residue (Fig. 6d, e). In contrast, compared with the vector control, FCoV-N expression was increased by approximately 60 % in cells transfected with wild-type *CypB* (Fig. 6e). Protein expression levels of endogenous *CypA* and *CypB* were not affected in the cells transfected with each plasmid (Fig. 6d). Although the number of virus copies in the cells transfected with wild-type *CypB* was increased, those in all cells transfected with the mutant genes were reduced compared with the control cells (Fig. 6f). These data suggest that the isomerase active sites of CypB are implicated in viral protein expression.

## Discussion

The aim of this study was to clarify the role of Cyps in FCoV replication. Stable expression of CypA and CypB enhanced viral replication and protein expression. Furthermore, Cyp expression was inhibited in cells transfected with respective *Cyp*-specific shRNAs. FCoV replication and protein expression were restored in KD-CypA-fcwf and KD-CypB-fcwf cells transfected with wild-type CypA and CypB expression vectors. However, FCoV replication was not completely inhibited in KD-CypA-fcwf or KD-CypB-fcwf cells. Hypothetically, the low residual levels of CypA and CypB in these cells supported viral replication, or other Cyps or host factors compensated for the decreased activities of CypA and CypB. Consequently, we examined the role of CypA and CypB in FCoV replication using *CypA*-KO and *CypB* *-*KO cell lines. While we searched for other feline CypA paralog genes using Kyoto Encyclopedia of Genes and Genome software, we found one paralog sequence. Two CypA gene types were located on A2 and C2 genomes, respectively. There were four nucleotide differences, which translated into two different amino acid residues. With regard to CypB, no paralog sequence was found. However, the guide RNA sequence used for CypA in this study targeted the same sequence between those CypAs. These data suggest that both CypA and CypB play important roles in FCoV replication. However, RT-qPCR analysis showed that FCoV replication was not completely inhibited in *Cyps*-KO cells. These results suggest that other host factors compensated for activities of CypA and CypB, e.g. protein interacting with NIMA (Pin1), a member of the parvulin subfamily of PPIases, modulates FCoV propagation [18].

A previous study revealed that the PPIase activities of R55A, F60A and H126Q CypA mutants were <1 % compared with those of wild-type CypA [16]. The activities of these mutants were reduced by 190–1000-fold compared with those of wild-type. Although we did not examine whether all mutants lack PPIase activities in this study, all active sites are conserved in other species’ CypA. There is no conclusive proof that the CypA (R55A, F60A and H126Q) mutants inhibited FCoV replication by dominant-negative effects on PPIase activity. However, all three active sites drastically reduced binding ability with CsA as shown by moe software analysis. Our present findings suggest that CsA-binding ability of CypA is related with FCoV replication. In contrast, FCoV-N expression levels in fcwf-4 cells expressing wild-type CypA were not significantly different from those in cells transfected with the control vector (Fig. 6a). This conflicts with the data shown in Fig. 1(a) and raises the possibility that FCoV replication in fcwf-4 cells stably expressing wild-type *CypA* is affected to a greater extent than that in over-expressing cells.

For experiments using the R62A and F67A of CypB mutants, this can lead to a dramatic loss of PPIase activity, between 8 and 12 % of wild-type CypB isomerase activity, as reported by Carpentier *et al*. [17]. Unfortunately, it was not examined whether these mutants lack PPIase activities in this study as described by CypA mutants. Compared with wild-type CypB, both the R62A and the F67A mutants drastically reduce CsA-binding efficiency as shown by moe software analysis (Fig. S2). However, the F67A mutant protein was unstable in Fig. 6(d) as shown by moe software analysis (Fig. S2). Therefore, the inhibition effects of the mutant F67A in FCoV replication need to be examined in detail. These results suggest that CsA-binding ability of CypB is related to FCoV replication.

Cellular chaperones control the correct folding of host and viral proteins [19]. Cyps with PPIase activity catalyse protein folding [8, 20] and mediate the life cycles of human immunodeficiency virus type 1 and hepatitis C virus [19]. The RNA chaperone domain of the N protein of CoV encircles genomic RNA (gRNA) to form a helical ribonucleoprotein structure [21, 22]. The N protein may participate in the discontinuous transcription of subgenomic mRNAs (sgmRNAs) because depletion of N from the replicon inhibits the synthesis of sgmRNA but not that of gRNA [23]. DDX1 is a cellular RNA helicase of the DEAD box helicase family, and KD of DDX1 reduces quantities of longer infectious bronchitis virus viral RNAs [24]. Phosphorylation of the N protein allows recruitment of DDX1 to the phosphorylated-N-containing complex that facilitates template reading, thereby enabling the synthesis of longer sgmRNAs [24]. Because the N protein of SARS-CoV binds CypA and CypA is incorporated into SARS-CoV particles [25], CsA may act directly on CypA/N protein complexes to prevent CoV from synthesizing gRNA and sgmRNA. However, we have yet to determine whether CypA/N protein complexes are incorporated into virion particles.

The replication of human and animal CoVs requires different Cyps. For example, CypA and CypB do not facilitate SARS-CoV replication [9], and the replication of human HCoV-NL63 requires CypA but not CypB [14]. This is a contradiction to our data showing that both CypA and CypB are required for FCoV replication. However, we have no evidence that other animal CoVs require different Cyps in replication. The different requirement for Cyps in viral replication between human and animal CoVs may result from the binding affinity of viral proteins to Cyp domains. Furthermore, previous studies on human CoV were performed using KD systems but not KO systems *in vitro*.

Recent analysis of single-nucleotide polymorphism variants of human CypA that affect HCoV-229E replication indicates that HCoV-229E replication in cells transfected with expression vectors encoding the *CYPA* D66E, N106I and G96D mutants was abolished [12]. The mutated amino acid residues are located near the PPIase active site, which is consistent with our present data. The mutant proteins of *CypA* and *CypB* used in this study strikingly reduce the affinities between CsA and Cyps (Figs S1 and S2). These findings may indicate that those affinities between CsA and Cyps affect FCoV replication. It may be presumed that any host or viral factors bind CsA-binding regions of Cyps to contribute to FCoV replication. Namely, CsA might competitively bind the regions against those factors to inhibit FCoV replication. Further research on the role of CsA-binding regions of Cyps in FCoV replication would clarify the mechanism of inhibition of FCoV and other CoV replication by CsA.

In conclusion, we demonstrate that the active sites of Cyp PPIase are crucial for FCoV replication. Further studies are required to determine the molecular mechanism of the function of cellular Cyps that contribute to the efficient FCoV replication. PPIase inhibitors may act as drug alternatives for treating FIP and other CoV-induced diseases.

## Methods

### Cell culture, infection and virus titration

fcwf-4 (American Type Culture Collection) was maintained in Dulbecco’s modified Eagle’s medium (Sigma-Aldrich) containing 10 % (v/v) FBS (JRH, Nissui). FCoV (strain 79–1146) was a gift from Dr Tsutomu Hodatsu, Kitasato University, Japan, and was propagated in fcwf-4 cells. Viruses were purified using linear sucrose-gradient ultracentrifugation. Each cell line was infected with FCoV at a m.o.i. of 1. Whole-cell lysates, RNAs and culture fluids were collected 20 h after infection. FCoV titres were expressed as TCID_50_. The viral stocks were prepared by collecting the virus particles from the culture medium, and TCID_50_ of each viral preparation was determined by infection of 8 wells of fcwf-4 cells in 96-well plates, in triplicate, with 10-fold serial dilutions of each viral stock.

### Plasmids

The feline genes encoding CypA and CypB (GenBank accession nos. AB_588985 and AB_588986) were isolated from a cDNA library prepared from fcwf-4 cells by PCR. In brief, total RNA derived from fcwf-4 cells was extracted using Isogen (Nippon Gene), and a cDNA library was constructed using SuperScript III RNase H^-^ Reverse Transcriptase (Invitrogen). The 5′- and 3′-regions of *CypA* and *CypB* were cloned using the FirstChoice RLM-RACE Kit (Thermo Fisher Scientific). Genes encoding Cyp were cloned into the pEF6/Myc-His A vector (Invitrogen) that contains the blasticidin-resistance gene for selection of stable cell lines, and the vectors were termed pEF-Myc-CypA and pEF-Myc-CypB. Sequences of the PCR primers are shown in Table 1. For mutagenesis, we generated the isomerase active-site mutants and calcineurin-binding region mutants of feline CypA (CypA-R55A, CypA-F60A, CypA-H126Q), as described by Zydowsky *et al*. [16], and those of feline CypB (Cyp-B-R62A, CypB-F67A), as described by Carpentier *et al*. [17, 26]. We used the respective parental pEF-Myc-CypA and pEF-Myc-CypB vectors with the PrimeSTAR Mutagenesis Basal Kit (Takara-bio), as shown in Tables 1, S1 and S2. Vector sequences were confirmed by Big-dye terminator sequence analysis (Life Technologies). For genetic KO experiments with fcwf-4 cells, we constructed plasmids using CRISPR/Cas9 systems. In brief, we synthesized oligonucleotides to guide RNA to target *CypA* and *CypB* DNA (Table 1). These were sub-cloned into the vector, pSpCas9 (BB)−2A-Puro (pX459: Addgene). Sequences of all constructed plasmids were confirmed using the Big-Dye Terminator v1.1 Cycle Sequencing Kit (Life Technologies).

**Table 1.** Primer sequences used in this study

Primer	Sequence
F-CpA-FW BamHI	5′-CGGGATCCACCATGGTCAACCCCATCGTGTTTTTTGAC-3′
F-CpA-RV EcoRI	5′-GGAATTCGATTTGTCCACAGTCAGCAATGGTGA-3′
F-CpB-FW BamHI	5′-CGGGATCCACCATGCTGCGCCTTTCGGAACGG-3′
F-CpB-RV EcoRI	5′-CGGAATTCTTCCTTGGCAATGGCAAAGGG-3′
F-CpAmut FW R55A	5′-TTTCACGCCATTATCCCGGGATTTATG-3′
F-CpAmut RV R55A	5′-GATAATGGCGTGAAAGCAGGAACCTTT-3′
F-CpAmut FW F60A	5′-CCGGGAGCTATGTGCCAGGGTGGTGAC-3′
F-CpAmut RV F60A	5′-GCACATAGCTCCCGGGATAATGGCGTG-3′
F-CpAmut FW H126Q	5′-GGCAAGCAAGTGGTGTTTGGCATGGTG-3′
F-CpAmut RV H126Q	5′-CACCACTTGCTTGCCATCCAACCACTC-3′
F-CpBmut FW R62A	5′-TTCCATGCTGTGATCAAGGACTTCATG-3′
F-CpBmut RV R62A	5′-GATCACAGCATGGAATTTGCTGTCTTT3′
F-CpBmut FW F67A	5′-AAGGACGCCATGATCCAGGGTGGAGAC
F-CpBmut RV F67A	5′-GATCATGGCGTCCTTGATCACACGATG
Feline CypA-Cas9 FW	5′-CACCGGAGAAAGGATTTGGTTACAA-3′
Feline CypA-Cas9 RV	5′-AAACTTGTAACCAAATCCTTTCTCC-3′
Feline CypB-Cas9 FW	5′-CACCGTTCGCCGCCGCCCTCATCGT-3′
Feline CypB-Cas9 RV	5′-AAACACGATGAGGGCGGCGGCGAAC-3′

### Antibodies

We used the following primary antibodies for Western blot analysis: anti-CypB (Thermo Scientific), anti-CypA (Millipore), anti-cMyc (Wako Laboratory Chemicals), anti-glyceraldehyde 3-phosphate dehydrogenase (Millipore) and anti-β-actin (Sigma-Aldrich). Immune complexes were visualized using an anti-mouse IgG (Promega) or anti-rabbit IgG antibodies (Promega), each of which was conjugated to HRP.

### Western blot analysis

Cell membranes were disrupted using cell-lysis buffer [10 mM Tris/HCl, pH 7.8, 1 mM EDTA, 1 % (w/v) NP-40, 0.15 M NaCl] containing the Complete Mini reagent (Roche Diagnostics). Lysate proteins were resolved by subjecting them to electrophoresis through 12.5 % SuperSep gel (Wako Laboratory Chemicals). The proteins were electrophoretically transferred from the gels to Immobilon-P membranes (Millipore) that were previously treated with 5 % (w/v) skimmed dry milk for 1 h at room temperature to minimize nonspecific binding. Membranes were then incubated for 1 h with the primary antibodies. Immune complexes were visualized using an anti-mouse IgG (Promega) or anti-rabbit IgG antibodies (Promega), each of which was conjugated to HRP, followed by incubation with an enhanced chemiluminescence substrate (SuperSignal West Femto Maximum Sensitivity Substrate; Thermo Scientific), according to the manufacturer’s protocol. Signals were detected using the ImageQuant LAS 4000-mini Imaging System (GE Healthcare Life Sciences) and analysed by Multi Gauge Version 3.0 software (GE Healthcare Life Sciences).

### Transfection of plasmids and establishment of stable cell line expressing CypA, CypB

The fcwf-4 cell line was plated on a 10 cm diameter dish (Corning) 18 h before transfection. Cells were transfected with 5 µg DNA of wild-type *CypA* or wild-type *CypB* (pEF-Myc-CypA or pEF-Myc-CypB) using the X-tremeGENE HP DNA Transfection Regent (Roche Diagnostics), according to the manufacturer’s instructions. The transfected cells were selected for 2 weeks with 6 µg ml^−1^ blasticidin (Life Technology) at 24 h after transfection. The individual cell colonies selected with blasticidin were cloned and analysed for expression of the target proteins by Western blot.

### Establishment of KD cells and Western blot analysis

Human immunodeficiency virus type 1 vectors were used to express shRNAs. To establish KD cell lines, which stably expressed shRNAs, we used the sh-A161 (shRNA target sequence of *CypA*; AAGGGTTCCTGCTTTCTCAGA) and sh-B710 (shRNA target sequence of *CypB*; AAGGTGGAGAGCACCAAGACA [27]) vectors to suppress CypA and CypB expression, respectively. These were provided by Dr Hengli Tang (Florida State University). The sh-A161 and sh-B710 vectors were used to transfect into fcwf-4 cells in the presence of X-tremeGENE HP DNA Transfection Reagent. Transfected cells were selected by treating cells with 6 µg ml^−1^ puromycin (Sigma-Aldrich), 24 h post-transfection. Stably transfected cell lines were obtained from each colony isolated after a 3 week selection. The fewf-4 cell lines established with the sh-A161 vector were termed KD-CypA-fcwf cells, and those cell lines established with the sh-B710 vector were termed KD-CypB-fcwf cells. Inhibition of protein expression by shRNAs was estimated by Western blot. Those KD cells were infected with the FCoV 79–1146 strain at a m.o.i. of 1 p.f.u. per cell to study the effects on FCoV infection before cells were collected for Western blot and RT-qPCR analyses at 20 h post-infection (p.i.).

### Reverse transcriptase-quantitative PCR (RT-qPCR)

The number of copies of viral genomes in fcwf-4 cells infected with FCoV was determined using RT-qPCR [11]. In brief, the medium was removed 20 h after infection, and RNA from the cells was prepared using Isogen (Nippongene), according to the manufacturer’s protocol. Total RNA was reverse transcribed using the PrimeScript RT-PCR Kit (Perfect Real Time; Takara Bio). Viral cDNAs were quantified using qPCR with primers specific for the gene encoding FCoV-N (forward, 5′-TGGCCACACAGGGACAAC-3′; reverse, 5′-AGAACGACCACGTCTTTTGGAA-3′) and the TaqMan probe (FAM-TTCATCTCCCCAGTTGACG-BHQ-1). Reaction mixtures were prepared according to the manufacturer’s protocol using Premix EXTaq (Takara Bio), and sequences were amplified using the 7500 Sequence Detection System (Life Technologies). The viral RNA copy number was normalized to that of the feline gene encoding feline β2-microglobulin (β2M) (GenBank accession no. NM_001009876) using qPCR with forward (5′-CGCGTTTTGTGGTCTTGGTCTTGGT-3′) and reverse (5′-AAACCTGAACCTTTGGAGAATGC-3′) primers specific for *β2M*. The TaqMan probe, TAMRA-CGGACTGCTCTATCTGTCCCACCTGGA-BHQ-2, was used to detect *β2M* mRNA.

### Establishment of KD-CypA and KD-CypB cells stably transfected with wild-type CypA and CypB expression vectors

KD-CypA-fcwf cells and KD-CypB-fcwf cells were plated on 10 cm dishes (Corning) and transfected with 5 µg DNA of pEF-Myc-CypA and pEF-Myc-CypB, respectively, using the X-tremeGENE HP DNA Transfection Reagent according to the manufacturer’s protocol. The transfected cells were selected with both 6 µg ml^−1^ blasticidin and 6 µg ml^−1^ puromycin at 24 h after transfection. The individual cell colonies selected with blasticidin and puromycin were cloned and analysed for expression of the target proteins by Western blot. Those KD cells restored with *CypA* or *CypB* were infected with FCoV 79–1146 at a m.o.i. of 1 p.f.u. per cell to study the effects on FCoV infection before cells were collected for Western blot and RT-qPCR analyses at 20 h p.i.

### KO of *CypA* or *CypB* in the fcwf-4 cells by CRISPR/Cas9 and virus infection

Plasmids constructed by CRISPR/Cas9 systems were transfected into fcwf-4 cells with X-tremeGENE HP DNA Transfection Reagent. More than 12 cell lines were cloned after selection with puromycin (6 µg ml^−1^) over a 2 week period. Mutations of each cell line were confirmed by Western blot and genomic DNA sequence analyses. Those KO cells were infected with FCoV 79–1146 strain at a m.o.i. of 1 to study the effects on FCoV infection before the cells were collected for Western blot and RT-qPCR analyses at 20 h p.i.

### Cells expressing CypA and CypB dominant-negative mutants inhibit FCoV replication

Efforts to establish cell lines stably expressing dominant-negative CypA and CypB mutants were unsuccessful. Therefore, we moved to gain-of-function/overexpression studies to test for their impact on FCoV infection. fcwf-4 cells were transfected with vectors encoding dominant-negative mutation using the X-tremeGENE HP DNA Transfection Reagent, as described previously. The cells were infected with FCoV at a m.o.i. of 1 at 24 h post-transfection and subjected to Western blot and RT-qPCR analyses at 20 h p.i.

### Statistical analysis

Differences between variables were statistically evaluated using the Student’s *t*-test. The threshold of statistical significance was defined as *P*<0.05. Values are expressed as the mean±sd.

### Cell viability analysis

We assayed WST-8 to evaluate the viability of the cells transfected with each plasmid. The Cell Counting Kit-8 (Dojin Chemical) was used for analysis according to the manufacturer’s instructions.

### Genomic database analysis

We used the Kyoto Encyclopedia of Genes and Genome to search for paralog genes of *Cyp*s on *F. catus* genomes.

### Protein affinity analysis

We used moe (Chemical Computing Group) to analyse the interaction between Cyps and CsA and protein stabilities.

## Supplementary Data

181 Supplementary File 1
