# Supplementary material for: Feline coronavirus replication is affected by both cyclophilin A and cyclophilin B
Source: J Gen Virol. 2017 Mar 13;98(2):190–200. doi: 10.1099/jgv.0.000663 (PMC7079567; doi:10.1099/jgv.0.000663)

Supplementary table 1: Amino acid sequences of dominant negative mutants of CypA

feline-CypA

-GSCFH**R**IIPG**F**MCQGGDFTRHNGTGGKSIYGEKFDDENFILKHTGPGILSMANAGPNTNGS

QFFICTAKTEWLDGK**H**VVFGM-

feline-CypA H126Q

-GSCFHRIIPGFMCQGGDFTRHNGTGGKSIYGEKFDDENFILKHTGPGILSMANAGPNTNGS

QFFICTAKTEWLDGK**Q**VVFGM-

feline-CypA R55A

-GSCFH**A**IIPGFMCQGGDFTRHNGTGGKSIYGEKFDDENFILKHTGPGILSMANAGPNTNGS

QFFICTAKTEWLDGKHVVFGM-

feline-CypA F60A

-GSCFHRIIPG**A**MCQGGDFTRHNGTGGKSIYGEKFDDENFILKHTGPGILSMANAGPNTNG

SQFFICTAKTEWLDGKHVVFGM-

Bold characters represent original and substituted amino acids respectively.

Supplementary table 2: Amino acid sequences of dominant negative mutants of CypB

**feline-CypB**
NSKFH**R**VIKD**F**MIQGGDFTRGDGTGGKSIYGERFPDENFKLKHYGPGWVSMANAGKDTNGSQFFITTVKTAWLDGKHV VFGKVLEGMEVVRKVESTKTDSRDKPLKDVIIADCGKIEVEKPFAIAKE
**feline-CypB R62A**
NSKFH**A**VIKDFMIQGGDFTRGDGTGGKSIYGERFPDENFKLKHYGPGWVSMANAGKDTNGSQFFITTVKTAWLDGKHV VFGKVLEGMEVVRKVESTKTDSRDKPLKDVIIADCGKIEVEKPFAIAKE
**feline-CypB F67A**
NSKFHRVIKD**A**MIQGGDFTAGDGTGGKSIYGERFPDENFKLKHYGPGWVSMANAGKDTNGSQFFITTVKTAWLDGKHV VFGKVLEGMEVVRKVESTKTDSRDKPLKDVIIADCGKIEVEKPFAIAKE

Bold characters represent original and substituted amino acids respectively.

Fig. S1. All three predicted PPIase-active sites used in this study reduce affinities between CypA and CsA as shown by MOE software analysis. (a) Computational inference of 3D-structure of feline CypA and CsA. (b) The larger number (kcal/mol) in reduced affinity (dAffinity) indicates more reduction of CsA-binding affinity with CypA. Affinity between CypA and CsA is reduced by point-mutations of CypA (CypA-R55A, CypA-R60A, and CypA- H126Q) as shown in dAfinity. Each amino acid residue substitutes an original amino acid residue for Ala. (c) Protein stability is decreased by point-mutations. The larger number (kcal/mol) in reduced stability (dStability) indicates more reduction in protein stability. (d) Blue polygonal line graph indicates affinity between CsA and CypA on each amino acid site. Red polygonal line graph shows reduction of protein stability on each amino acid site. The larger kcal/mol indicates more reduction of protein stability and affinity between CypA and CsA.

Fig. S2. Both predicted PPIase-active sites used in this study reduce affinities between CypB and CsA as shown by MOE software analysis. (a) Computational inference of 3D-structure of feline CypB and CsA. (b) The larger number (kcal/mol) in reduced affinity (dAffinity) indicates more reduction of CsA-binding affinity with CypB. Affinity between CypB and CsA is reduced by point-mutations of CypB (CypB-R62A

and F67A) as shown in dAfinity. Each amino acid residue substitutes an original amino acid residue for Ala. (c) Protein stability is decreased by point mutations. The larger number (kcal/mol) in reduced stability (dStability) indicates more reduction of protein stability. (d) Blue polygonal line graph indicates affinity between CsA and CypB on each amino acid site. Red polygonal line graph shows reduction of protein stability on each amino acid site. The larger kcal/mol indicates more reduction of protein stability and affinity between CypB and CsA.

(a)

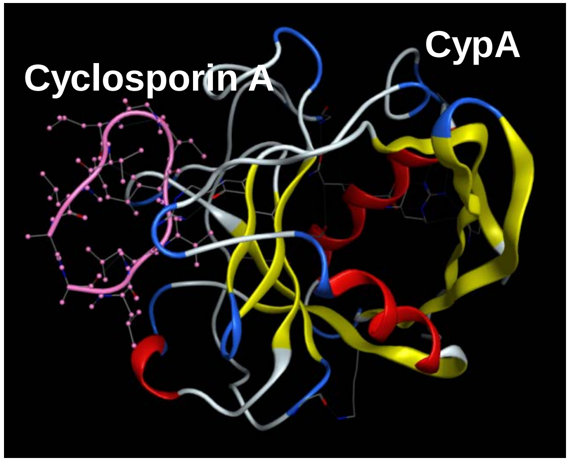

(b)

Affinity down top 10	
Site number	dAffinity
122	8.2506
126	8.1432
60	6.29
55	5.8813
121	4.4463
111	3.1672
150	3.0488
125	2.6268
58	1.7697
61	1.4172

(c)

Stability down top 10	
Site number	dStability
121	5.0075
113	4.3459
22	4.3184
48	4.2729
7	4.252
83	4.2232
60	4.1833
53	4.057
112	4.0443
129	3.9625

(d)

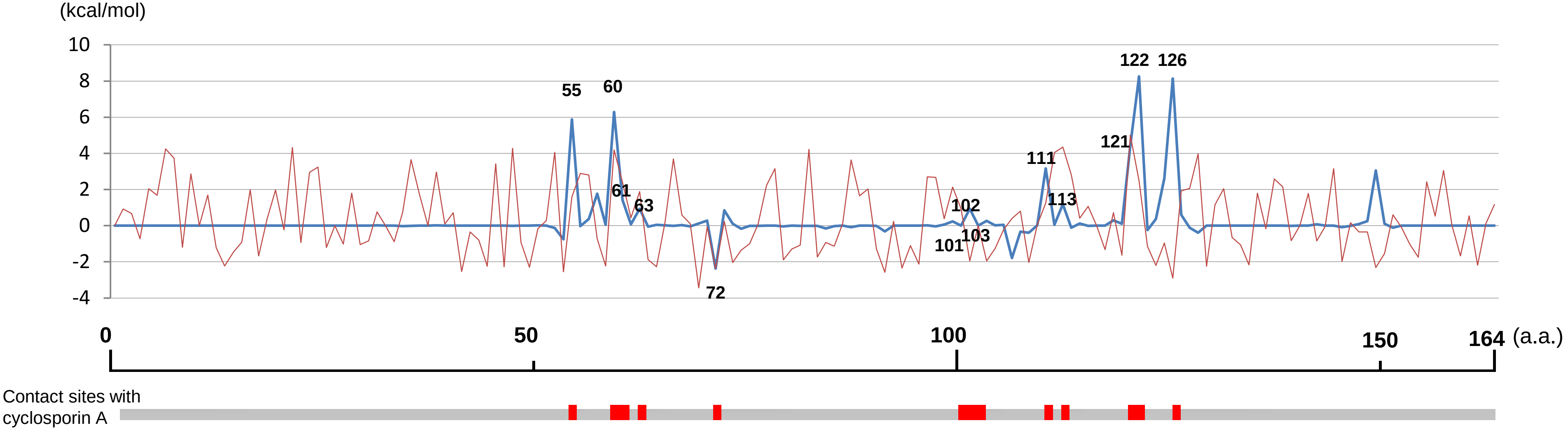

(a)

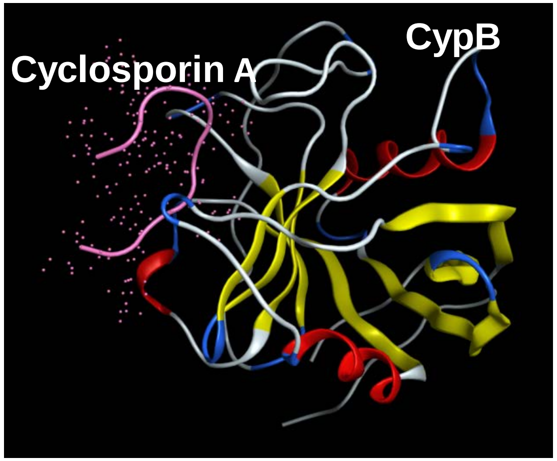

(b)

Affinity down top 10

Site number	dAffinity
129	9.2771
133	6.5493
67	6.4817
62	6.3549
128	4.4601
118	3.3091
159	2.4897
120	1.8404
68	1.7758
89	1.1906

(c)

Stability down top 10

Site number	dStability
104	5.4276
127	5.0325
120	4.531
67	4.4057
136	4.1882
32	4.1864
119	4.1847
55	4.1721
90	3.9242
15	3.8377

(d)

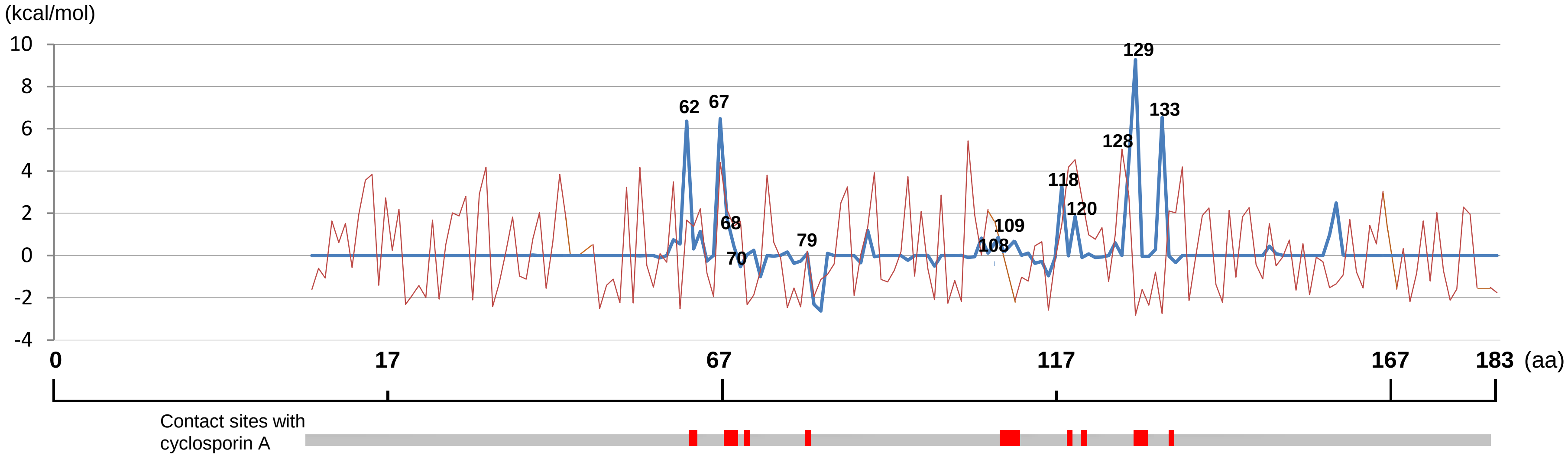

Supplement: Supplementary file 1 [file jgv-98-190-s001.pdf]
